# Triethyl­ammonium 1,1′-binaphthyl-2,2′-diyl phosphate

**DOI:** 10.1107/S160053681002026X

**Published:** 2010-06-16

**Authors:** Ravikumar R. Gowda, Venkatachalam Ramkumar, Debashis Chakraborty

**Affiliations:** a Department of Chemistry, IIT Madras, Chennai, TamilNadu, India

## Abstract

In the crystal structure of the title compound, C_6_H_16_N^+^·C_20_H_12_O_4_P^−^, an N—H⋯O inter­action links the cation to the anion. The N atom in the triethyl­ammonium cation exhibits a trigonal-bipyramidal coordination geometry and forms an N—H⋯O inter­action with one phosphate O atom of the 1,1′-binaphthyl-2,2′-diyl phosphate ligand. A bifurcated C—H⋯O inter­action with the other phosphate O atom links molecules along the *a* axis. The dihedral angle between the two naphthyl ring systems is 58.92 (3)°. The refined Flack parameter value of 0.50 (10) indicates inversion twinning.

## Related literature

For the use of binolphospho­ric acid in synthesis, see: Jacques *et al.* (1971 ▶); Moreau *et al.*, (2009 ▶). For the binaphthyl unit in host compounds, see: Kyba *et al.* (1977 ▶). 
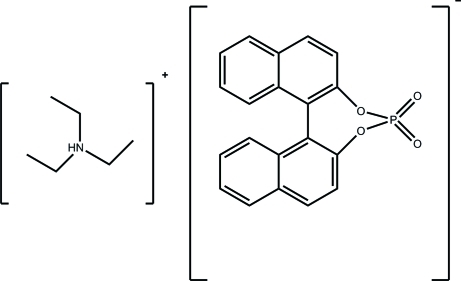


## Experimental

### 

#### Crystal data

C_6_H_16_N^+^·C_20_H_12_O_4_P^−^
*M* *_r_* = 449.46
Orthorhombic, 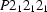

*a* = 8.4605 (2) Å
*b* = 13.3603 (4) Å
*c* = 20.5688 (7) Å
*V* = 2324.99 (12) Å^3^
*Z* = 4
Mo *K*α radiation
μ = 0.15 mm^−1^
*T* = 298 K
0.32 × 0.27 × 0.22 mm

#### Data collection

Bruker APEXII CCD area-detector diffractometer
Absorption correction: multi-scan (*SADABS*; Bruker, 1999 ▶) *T*_min_ = 0.953, *T*_max_ = 0.968
30327 measured reflections
5561 independent reflections
4823 reflections with *I* > 2σ(*I*)
*R*_int_ = 0.028

#### Refinement

*R*[*F* ^2^ > 2σ(*F* ^2^)] = 0.043
*wR*(*F* ^2^) = 0.121
*S* = 1.04
5561 reflections
297 parameters
H atoms treated by a mixture of independent and constrained refinement
Δρ_max_ = 0.54 e Å^−3^
Δρ_min_ = −0.31 e Å^−3^
Absolute structure: Flack (1983 ▶)
Flack parameter: 0.50 (10)

### 

Data collection: *APEX2* (Bruker, 2004 ▶); cell refinement: *APEX2* and *SAINT-Plus* (Bruker, 2004 ▶); data reduction: *SAINT-Plus* and *XPREP* (Bruker, 2004 ▶); program(s) used to solve structure: *SHELXS97* (Sheldrick, 2008 ▶); program(s) used to refine structure: *SHELXL97* (Sheldrick, 2008 ▶); molecular graphics: *ORTEP-3* (Farrugia, 1997 ▶); software used to prepare material for publication: *SHELXL97*.

## Supplementary Material

Crystal structure: contains datablocks global, I. DOI: 10.1107/S160053681002026X/bq2208sup1.cif

Structure factors: contains datablocks I. DOI: 10.1107/S160053681002026X/bq2208Isup2.hkl

Additional supplementary materials:  crystallographic information; 3D view; checkCIF report

## Figures and Tables

**Table 1:** Hydrogen-bond geometry (Å, °)

*D*—H⋯*A*	*D*—H	H⋯*A*	*D*⋯*A*	*D*—H⋯*A*
N1—H1*N*⋯O4	0.87 (3)	1.83 (3)	2.689 (2)	172 (2)
C24—H24*B*⋯O3^i^	0.97	2.47	3.342 (4)	149
C26—H26*A*⋯O3^i^	0.97	2.47	3.373 (3)	155

Symmetry code: (i) 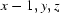.

**Fig. 1.**
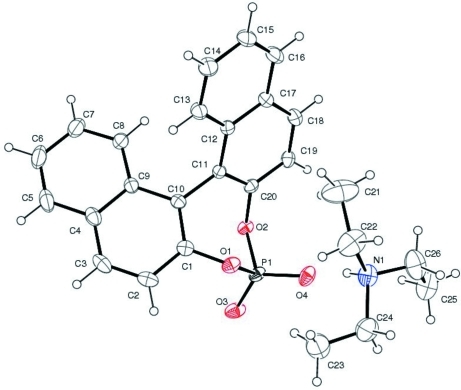
The ORTEP drawing of the molecule with atoms represented as 30% probability ellipsoids.

**Fig. 2.**
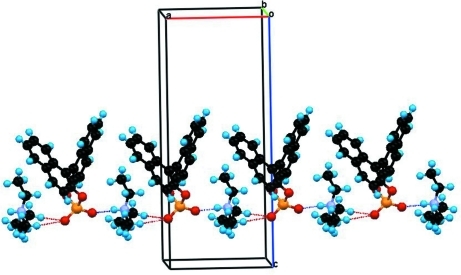
The Packing diagram showing the N-H···O (blue dashed line) and the bifurcated C-H···O interactions (red dashed line)

## supplementary crystallographic information

### Comment

The title compound is a salt of binol phosphoric acid. It represents a useful tool for the resolution of amines. Amines which are unable to resolve using other chiral acids, are resolved using binolphosphoric acid very easily and in high yield (Jacques *et al.*, 1971). Optically active amines are useful as intermediates of medicines, agricultural chemicals, or the like can be produced without special post-treatment in high yield at high optical purity using optically active phosphoric acid derivatives. A recent report depicts phosphoric acid acts as Bronsted acid to catalyze the addition of enolizable β-diketones, β-ketoesters, and vinylogous amides to α,β-unsaturated aldehydes to lead to substituted chromenones, pyranones, and tetra hydroquinolinones in good yields under mild reaction conditions via a formal [3+3] cycloaddition (Moreau *et al.*, 2009).

In the title compound  C_26_H_28_N O_4_P, (I), the 1,1'-binaphthyl-2-2'diyl phosphate ligand coordinates with the triethyl ammonium to form an intra molecular N-H..O interaction with one phosphate O atom and with another phosphate O atom with which a bifurcated C-H..O interaction (Table 1) along the *a* axis extending into a network (Figure 2). The molecular structure viewed down along the C10-C11 pivot, clearly shows the non co-planar geometry of the two naptha rings system with a dihedral angle of 58.92 (3)°.

### Experimental

To a stirred ice cold solution of 0.2 g (0.69 mole) binol (Evan *et. al*, 1977) in 20 mL of dichloromethane under nitrogen atmosphere was added 0.07 mL (0.69 mmol) POCl_3_ drop wise followed by addition of 0.5 mL (3.5 mmol) triethylamine. White fumes of HCl were observed upon addition, reaction mixture was stirred at 0 °C for 30 minutes. Then 0.13 mL (6.9 mmol) H_2_O was added slowly at 0 °C. Reaction mixture was stirred at 0 °C for 1 h and warmed up to room temperature and stirred for 40 h. The reaction was monitored using thin layer chromatography. The product was extracted using dichloromethane and purified by crystallization in dichloromethane. Yield is found to be 0.26 g (83.9 %).

### Refinement

All hydrogen atoms were fixed geometrically and allowed to ride on the parent carbon atoms with aromatic C-H = 0.93 Å, aliphatic C-H = 0.98 Å and methyl C-H = 0.96 Å. The displacement parameters were set for phenyl and aliphatic H atoms at *U*_iso_(H) = 1.2*U*_eq_(C) and for methyl H atoms at *U*_iso_(H) = 1.5*U*_eq_(C). The Flack parameter was refined as a full least-squares variable, and the refined value of 0.50 (10) suggests inversion twinning.

### Crystal data

**Table:**

C_6_H_16_N^+^·C_20_H_12_O_4_P^−^	*F*(000) = 952
*M**_r_* = 449.46	*D*_x_ = 1.284 Mg m^−^^3^
Orthorhombic, *P*2_1_2_1_2_1_	Mo *K*α radiation, λ = 0.71073 Å
Hall symbol: P 2ac 2ab	Cell parameters from 7503 reflections
*a* = 8.4605 (2) Å	θ = 2.5–27.9°
*b* = 13.3603 (4) Å	µ = 0.15 mm^−^^1^
*c* = 20.5688 (7) Å	*T* = 298 K
*V* = 2324.99 (12) Å^3^	Block, white
*Z* = 4	0.32 × 0.27 × 0.22 mm

### Data collection

**Table:**

Bruker APEXII CCD area-detector diffractometer	5561 independent reflections
Radiation source: fine-focus sealed tube	4823 reflections with *I* > 2σ(*I*)
graphite	*R*_int_ = 0.028
phi and ω scans	θ_max_ = 28.3°, θ_min_ = 2.5°
Absorption correction: multi-scan (*SADABS*; Bruker, 1999)	*h* = −10→10
*T*_min_ = 0.953, *T*_max_ = 0.968	*k* = −17→15
30327 measured reflections	*l* = −27→27

### Refinement

**Table:**

Refinement on *F*^2^	Hydrogen site location: inferred from neighbouring sites
Least-squares matrix: full	H atoms treated by a mixture of independent and constrained refinement
*R*[*F*^2^ > 2σ(*F*^2^)] = 0.043	*w* = 1/[σ^2^(*F*_o_^2^) + (0.0768*P*)^2^ + 0.3083*P*] where *P* = (*F*_o_^2^ + 2*F*_c_^2^)/3
*wR*(*F*^2^) = 0.121	(Δ/σ)_max_ < 0.001
*S* = 1.04	Δρ_max_ = 0.54 e Å^−^^3^
5561 reflections	Δρ_min_ = −0.30 e Å^−^^3^
297 parameters	Extinction correction: *SHELXL97* (Sheldrick, 2008), Fc^*^=kFc[1+0.001xFc^2^λ^3^/sin(2θ)]^-1/4^
0 restraints	Extinction coefficient: 0.000
Primary atom site location: structure-invariant direct methods	Absolute structure: Flack (1983)
Secondary atom site location: difference Fourier map	Flack parameter: 0.50 (10)

### Special details

**Table:**

Geometry. All esds (except the esd in the dihedral angle between two l.s. planes) are estimated using the full covariance matrix. The cell esds are taken into account individually in the estimation of esds in distances, angles and torsion angles; correlations between esds in cell parameters are only used when they are defined by crystal symmetry. An approximate (isotropic) treatment of cell esds is used for estimating esds involving l.s. planes.
Refinement. Refinement of F^2^ against ALL reflections. The weighted R-factor wR and goodness of fit S are based on F^2^, conventional R-factors R are based on F, with F set to zero for negative F^2^. The threshold expression of F^2^ > 2sigma(F^2^) is used only for calculating R-factors(gt) etc. and is not relevant to the choice of reflections for refinement. R-factors based on F^2^ are statistically about twice as large as those based on F, and R- factors based on ALL data will be even larger.

### Fractional atomic coordinates and isotropic or equivalent isotropic displacement parameters (Å^2^)

**Table:**

	*x*	*y*	*z*	*U*_iso_*/*U*_eq_	
C1	0.9569 (2)	−0.09228 (15)	0.66118 (9)	0.0337 (4)	
C2	1.0264 (3)	−0.18489 (16)	0.67555 (11)	0.0431 (5)	
H2	0.9953	−0.2203	0.7123	0.052*	
C3	1.1397 (3)	−0.22298 (15)	0.63547 (12)	0.0475 (5)	
H3	1.1837	−0.2853	0.6444	0.057*	
C4	1.1909 (3)	−0.16849 (15)	0.58042 (10)	0.0423 (5)	
C5	1.3154 (3)	−0.2041 (2)	0.54033 (13)	0.0600 (7)	
H5	1.3603	−0.2662	0.5489	0.072*	
C6	1.3702 (4)	−0.1483 (2)	0.48930 (14)	0.0677 (8)	
H6	1.4518	−0.1728	0.4635	0.081*	
C7	1.3040 (3)	−0.0549 (2)	0.47599 (11)	0.0546 (6)	
H7	1.3435	−0.0165	0.4419	0.065*	
C8	1.1824 (3)	−0.01962 (17)	0.51244 (9)	0.0410 (5)	
H8	1.1381	0.0421	0.5021	0.049*	
C9	1.1211 (2)	−0.07409 (14)	0.56575 (9)	0.0323 (4)	
C10	0.9960 (2)	−0.03785 (14)	0.60657 (8)	0.0298 (4)	
C11	0.9160 (2)	0.05934 (13)	0.59444 (8)	0.0283 (4)	
C12	0.8330 (2)	0.07958 (14)	0.53500 (8)	0.0309 (4)	
C13	0.8099 (3)	0.00551 (16)	0.48651 (10)	0.0408 (5)	
H13	0.8506	−0.0584	0.4928	0.049*	
C14	0.7289 (3)	0.0268 (2)	0.43082 (10)	0.0518 (6)	
H14	0.7143	−0.0229	0.3997	0.062*	
C15	0.6676 (3)	0.1229 (2)	0.42019 (11)	0.0576 (7)	
H15	0.6153	0.1371	0.3815	0.069*	
C16	0.6841 (3)	0.19519 (19)	0.46587 (11)	0.0506 (6)	
H16	0.6415	0.2583	0.4585	0.061*	
C17	0.7658 (3)	0.17582 (15)	0.52499 (9)	0.0368 (4)	
C18	0.7767 (3)	0.24789 (15)	0.57516 (10)	0.0432 (5)	
H18	0.7358	0.3116	0.5684	0.052*	
C19	0.8456 (2)	0.22598 (15)	0.63296 (10)	0.0368 (4)	
H19	0.8485	0.2734	0.6660	0.044*	
C20	0.9126 (2)	0.13082 (14)	0.64237 (8)	0.0291 (4)	
C21	0.4505 (5)	0.0741 (4)	0.64124 (17)	0.1067 (15)	
H21A	0.5583	0.0913	0.6504	0.160*	
H21B	0.3883	0.1340	0.6381	0.160*	
H21C	0.4454	0.0383	0.6008	0.160*	
C22	0.3853 (4)	0.0076 (3)	0.69635 (16)	0.0853 (10)	
H22A	0.2711	0.0039	0.6928	0.102*	
H22B	0.4271	−0.0597	0.6917	0.102*	
C23	0.4903 (4)	−0.1247 (2)	0.8033 (2)	0.0832 (10)	
H23A	0.5959	−0.1078	0.7903	0.125*	
H23B	0.4408	−0.1636	0.7698	0.125*	
H23C	0.4936	−0.1629	0.8428	0.125*	
C24	0.3995 (4)	−0.0330 (2)	0.81405 (17)	0.0715 (8)	
H24A	0.4278	−0.0052	0.8560	0.086*	
H24B	0.2879	−0.0494	0.8152	0.086*	
C25	0.4079 (4)	0.1960 (3)	0.83499 (17)	0.0788 (9)	
H25A	0.5187	0.2078	0.8285	0.118*	
H25B	0.3927	0.1586	0.8744	0.118*	
H25C	0.3536	0.2589	0.8382	0.118*	
C26	0.3437 (3)	0.1379 (3)	0.77885 (19)	0.0791 (9)	
H26A	0.2352	0.1200	0.7887	0.095*	
H26B	0.3415	0.1816	0.7412	0.095*	
N1	0.4281 (2)	0.04695 (16)	0.76106 (11)	0.0490 (5)	
O1	0.84192 (17)	−0.05577 (10)	0.70278 (6)	0.0372 (3)	
O2	0.98292 (15)	0.11072 (10)	0.70184 (6)	0.0308 (3)	
O3	1.01033 (17)	0.00302 (13)	0.79917 (7)	0.0491 (4)	
O4	0.74028 (17)	0.08038 (13)	0.77268 (7)	0.0451 (4)	
P1	0.89188 (6)	0.03523 (4)	0.75172 (2)	0.03329 (13)	
H1N	0.529 (4)	0.0593 (18)	0.7609 (12)	0.044 (7)*	

### Atomic displacement parameters (Å^2^)

**Table:**

	*U*^11^	*U*^22^	*U*^33^	*U*^12^	*U*^13^	*U*^23^
C1	0.0328 (10)	0.0342 (10)	0.0342 (9)	−0.0053 (8)	−0.0050 (8)	−0.0002 (7)
C2	0.0506 (13)	0.0361 (10)	0.0424 (10)	−0.0078 (10)	−0.0115 (10)	0.0096 (9)
C3	0.0560 (15)	0.0293 (10)	0.0573 (12)	0.0071 (10)	−0.0186 (11)	0.0003 (9)
C4	0.0451 (13)	0.0371 (11)	0.0446 (11)	0.0090 (9)	−0.0150 (10)	−0.0086 (8)
C5	0.0611 (17)	0.0540 (14)	0.0649 (16)	0.0286 (13)	−0.0112 (13)	−0.0187 (12)
C6	0.0567 (18)	0.090 (2)	0.0565 (15)	0.0292 (15)	0.0067 (13)	−0.0209 (14)
C7	0.0493 (14)	0.0724 (17)	0.0421 (12)	0.0084 (12)	0.0076 (10)	−0.0062 (10)
C8	0.0397 (12)	0.0492 (12)	0.0341 (9)	0.0076 (9)	0.0003 (8)	−0.0012 (8)
C9	0.0336 (10)	0.0330 (9)	0.0303 (8)	0.0042 (8)	−0.0060 (7)	−0.0057 (7)
C10	0.0314 (10)	0.0295 (8)	0.0285 (8)	−0.0011 (8)	−0.0059 (7)	−0.0013 (7)
C11	0.0272 (9)	0.0312 (9)	0.0266 (8)	0.0016 (7)	0.0007 (7)	−0.0001 (6)
C12	0.0300 (10)	0.0345 (9)	0.0281 (8)	0.0037 (8)	−0.0001 (7)	−0.0002 (7)
C13	0.0452 (13)	0.0416 (11)	0.0355 (10)	0.0086 (9)	−0.0082 (9)	−0.0055 (8)
C14	0.0579 (15)	0.0613 (14)	0.0363 (10)	0.0111 (12)	−0.0154 (10)	−0.0122 (10)
C15	0.0617 (16)	0.0760 (17)	0.0351 (11)	0.0244 (14)	−0.0169 (11)	−0.0014 (11)
C16	0.0550 (15)	0.0526 (13)	0.0443 (12)	0.0177 (11)	−0.0136 (11)	0.0031 (10)
C17	0.0364 (11)	0.0393 (10)	0.0347 (9)	0.0072 (9)	−0.0023 (8)	0.0025 (8)
C18	0.0501 (13)	0.0339 (10)	0.0456 (11)	0.0124 (9)	0.0008 (10)	0.0005 (8)
C19	0.0387 (11)	0.0341 (9)	0.0375 (9)	0.0034 (8)	0.0028 (8)	−0.0092 (8)
C20	0.0251 (9)	0.0348 (9)	0.0275 (8)	−0.0043 (7)	0.0019 (7)	−0.0002 (7)
C21	0.106 (3)	0.148 (4)	0.066 (2)	−0.055 (3)	−0.031 (2)	0.016 (2)
C22	0.0621 (19)	0.124 (3)	0.0700 (19)	−0.028 (2)	−0.0083 (16)	−0.0116 (19)
C23	0.066 (2)	0.0684 (19)	0.115 (3)	−0.0038 (17)	0.019 (2)	0.0201 (19)
C24	0.0518 (16)	0.081 (2)	0.0814 (19)	−0.0096 (16)	0.0100 (15)	0.0077 (16)
C25	0.067 (2)	0.080 (2)	0.090 (2)	−0.0061 (17)	0.0116 (18)	−0.0214 (18)
C26	0.0399 (14)	0.0747 (19)	0.123 (3)	−0.0043 (14)	−0.0115 (17)	−0.0150 (19)
N1	0.0287 (10)	0.0563 (11)	0.0620 (12)	−0.0120 (8)	−0.0041 (8)	−0.0065 (9)
O1	0.0352 (7)	0.0421 (8)	0.0344 (7)	−0.0118 (6)	0.0018 (6)	0.0011 (5)
O2	0.0270 (7)	0.0402 (7)	0.0253 (6)	−0.0071 (6)	−0.0016 (5)	−0.0022 (5)
O3	0.0333 (8)	0.0790 (11)	0.0349 (7)	−0.0104 (8)	−0.0052 (6)	0.0127 (7)
O4	0.0269 (7)	0.0676 (10)	0.0408 (7)	−0.0058 (7)	0.0043 (6)	−0.0114 (7)
P1	0.0237 (2)	0.0516 (3)	0.0245 (2)	−0.0080 (2)	0.00039 (18)	−0.0002 (2)

### Geometric parameters (Å, °)

**Table:**

C1—C10	1.378 (3)	C18—C19	1.356 (3)
C1—O1	1.385 (2)	C18—H18	0.9300
C1—C2	1.401 (3)	C19—C20	1.405 (3)
C2—C3	1.363 (4)	C19—H19	0.9300
C2—H2	0.9300	C20—O2	1.386 (2)
C3—C4	1.414 (3)	C21—C22	1.542 (5)
C3—H3	0.9300	C21—H21A	0.9600
C4—C5	1.419 (3)	C21—H21B	0.9600
C4—C9	1.425 (3)	C21—H21C	0.9600
C5—C6	1.368 (4)	C22—N1	1.476 (4)
C5—H5	0.9300	C22—H22A	0.9700
C6—C7	1.396 (4)	C22—H22B	0.9700
C6—H6	0.9300	C23—C24	1.463 (4)
C7—C8	1.358 (3)	C23—H23A	0.9600
C7—H7	0.9300	C23—H23B	0.9600
C8—C9	1.415 (3)	C23—H23C	0.9600
C8—H8	0.9300	C24—N1	1.545 (4)
C9—C10	1.435 (3)	C24—H24A	0.9700
C10—C11	1.485 (3)	C24—H24B	0.9700
C11—C20	1.373 (2)	C25—C26	1.494 (4)
C11—C12	1.436 (2)	C25—H25A	0.9600
C12—C13	1.418 (3)	C25—H25B	0.9600
C12—C17	1.421 (3)	C25—H25C	0.9600
C13—C14	1.365 (3)	C26—N1	1.456 (4)
C13—H13	0.9300	C26—H26A	0.9700
C14—C15	1.402 (3)	C26—H26B	0.9700
C14—H14	0.9300	N1—H1N	0.87 (3)
C15—C16	1.355 (3)	O1—P1	1.6340 (14)
C15—H15	0.9300	O2—P1	1.6319 (13)
C16—C17	1.422 (3)	O3—P1	1.4636 (15)
C16—H16	0.9300	O4—P1	1.4815 (16)
C17—C18	1.414 (3)		
			
C10—C1—O1	119.10 (18)	C18—C19—H19	120.3
C10—C1—C2	122.5 (2)	C20—C19—H19	120.3
O1—C1—C2	118.39 (18)	C11—C20—O2	119.33 (16)
C3—C2—C1	119.8 (2)	C11—C20—C19	122.59 (17)
C3—C2—H2	120.1	O2—C20—C19	118.03 (16)
C1—C2—H2	120.1	C22—C21—H21A	109.5
C2—C3—C4	120.50 (19)	C22—C21—H21B	109.5
C2—C3—H3	119.7	H21A—C21—H21B	109.5
C4—C3—H3	119.7	C22—C21—H21C	109.5
C3—C4—C5	121.3 (2)	H21A—C21—H21C	109.5
C3—C4—C9	119.9 (2)	H21B—C21—H21C	109.5
C5—C4—C9	118.8 (2)	N1—C22—C21	111.7 (3)
C6—C5—C4	121.0 (2)	N1—C22—H22A	109.3
C6—C5—H5	119.5	C21—C22—H22A	109.3
C4—C5—H5	119.5	N1—C22—H22B	109.3
C5—C6—C7	120.1 (2)	C21—C22—H22B	109.3
C5—C6—H6	120.0	H22A—C22—H22B	107.9
C7—C6—H6	120.0	C24—C23—H23A	109.5
C8—C7—C6	120.4 (3)	C24—C23—H23B	109.5
C8—C7—H7	119.8	H23A—C23—H23B	109.5
C6—C7—H7	119.8	C24—C23—H23C	109.5
C7—C8—C9	121.8 (2)	H23A—C23—H23C	109.5
C7—C8—H8	119.1	H23B—C23—H23C	109.5
C9—C8—H8	119.1	C23—C24—N1	113.0 (2)
C8—C9—C4	117.86 (19)	C23—C24—H24A	109.0
C8—C9—C10	123.39 (17)	N1—C24—H24A	109.0
C4—C9—C10	118.73 (18)	C23—C24—H24B	109.0
C1—C10—C9	118.40 (17)	N1—C24—H24B	109.0
C1—C10—C11	119.26 (17)	H24A—C24—H24B	107.8
C9—C10—C11	122.20 (16)	C26—C25—H25A	109.5
C20—C11—C12	118.06 (16)	C26—C25—H25B	109.5
C20—C11—C10	119.80 (15)	H25A—C25—H25B	109.5
C12—C11—C10	122.05 (16)	C26—C25—H25C	109.5
C13—C12—C17	118.33 (17)	H25A—C25—H25C	109.5
C13—C12—C11	122.32 (17)	H25B—C25—H25C	109.5
C17—C12—C11	119.31 (17)	N1—C26—C25	116.7 (3)
C14—C13—C12	120.9 (2)	N1—C26—H26A	108.1
C14—C13—H13	119.5	C25—C26—H26A	108.1
C12—C13—H13	119.5	N1—C26—H26B	108.1
C13—C14—C15	120.5 (2)	C25—C26—H26B	108.1
C13—C14—H14	119.8	H26A—C26—H26B	107.3
C15—C14—H14	119.8	C26—N1—C22	113.8 (3)
C16—C15—C14	120.4 (2)	C26—N1—C24	108.8 (2)
C16—C15—H15	119.8	C22—N1—C24	110.6 (2)
C14—C15—H15	119.8	C26—N1—H1N	108.9 (16)
C15—C16—C17	120.9 (2)	C22—N1—H1N	107.8 (17)
C15—C16—H16	119.6	C24—N1—H1N	106.7 (16)
C17—C16—H16	119.6	C1—O1—P1	117.46 (12)
C18—C17—C12	118.96 (17)	C20—O2—P1	118.16 (11)
C18—C17—C16	122.1 (2)	O3—P1—O4	121.23 (9)
C12—C17—C16	118.87 (19)	O3—P1—O2	106.13 (8)
C19—C18—C17	121.39 (18)	O4—P1—O2	109.87 (9)
C19—C18—H18	119.3	O3—P1—O1	111.67 (9)
C17—C18—H18	119.3	O4—P1—O1	104.96 (8)
C18—C19—C20	119.30 (18)	O2—P1—O1	101.22 (7)
			
C10—C1—C2—C3	1.5 (3)	C12—C13—C14—C15	0.5 (4)
O1—C1—C2—C3	179.86 (19)	C13—C14—C15—C16	−1.8 (4)
C1—C2—C3—C4	1.8 (3)	C14—C15—C16—C17	1.0 (4)
C2—C3—C4—C5	176.2 (2)	C13—C12—C17—C18	174.9 (2)
C2—C3—C4—C9	−1.5 (3)	C11—C12—C17—C18	−2.7 (3)
C3—C4—C5—C6	−176.3 (2)	C13—C12—C17—C16	−2.6 (3)
C9—C4—C5—C6	1.4 (4)	C11—C12—C17—C16	179.8 (2)
C4—C5—C6—C7	0.1 (4)	C15—C16—C17—C18	−176.1 (3)
C5—C6—C7—C8	−1.6 (4)	C15—C16—C17—C12	1.3 (4)
C6—C7—C8—C9	1.7 (4)	C12—C17—C18—C19	−2.0 (3)
C7—C8—C9—C4	−0.2 (3)	C16—C17—C18—C19	175.4 (2)
C7—C8—C9—C10	178.0 (2)	C17—C18—C19—C20	2.3 (3)
C3—C4—C9—C8	176.4 (2)	C12—C11—C20—O2	175.82 (16)
C5—C4—C9—C8	−1.3 (3)	C10—C11—C20—O2	−0.6 (3)
C3—C4—C9—C10	−1.9 (3)	C12—C11—C20—C19	−6.7 (3)
C5—C4—C9—C10	−179.6 (2)	C10—C11—C20—C19	176.85 (18)
O1—C1—C10—C9	176.82 (16)	C18—C19—C20—C11	2.2 (3)
C2—C1—C10—C9	−4.8 (3)	C18—C19—C20—O2	179.68 (19)
O1—C1—C10—C11	1.1 (3)	C25—C26—N1—C22	−165.1 (3)
C2—C1—C10—C11	179.47 (18)	C25—C26—N1—C24	71.1 (3)
C8—C9—C10—C1	−173.24 (18)	C21—C22—N1—C26	71.5 (4)
C4—C9—C10—C1	4.9 (3)	C21—C22—N1—C24	−165.7 (3)
C8—C9—C10—C11	2.3 (3)	C23—C24—N1—C26	−175.3 (3)
C4—C9—C10—C11	−179.53 (17)	C23—C24—N1—C22	59.1 (4)
C1—C10—C11—C20	52.0 (3)	C10—C1—O1—P1	−76.8 (2)
C9—C10—C11—C20	−123.5 (2)	C2—C1—O1—P1	104.78 (18)
C1—C10—C11—C12	−124.3 (2)	C11—C20—O2—P1	−74.93 (19)
C9—C10—C11—C12	60.2 (3)	C19—C20—O2—P1	107.47 (18)
C20—C11—C12—C13	−170.67 (19)	C20—O2—P1—O3	162.56 (13)
C10—C11—C12—C13	5.7 (3)	C20—O2—P1—O4	−64.72 (14)
C20—C11—C12—C17	6.8 (3)	C20—O2—P1—O1	45.86 (14)
C10—C11—C12—C17	−176.80 (18)	C1—O1—P1—O3	−65.43 (15)
C17—C12—C13—C14	1.7 (3)	C1—O1—P1—O4	161.44 (14)
C11—C12—C13—C14	179.3 (2)	C1—O1—P1—O2	47.12 (14)

### Hydrogen-bond geometry (Å, °)

**Table:**

*D*—H···*A*	*D*—H	H···*A*	*D*···*A*	*D*—H···*A*
N1—H1N···O4	0.87 (3)	1.83 (3)	2.689 (2)	172 (2)
C24—H24B···O3^i^	0.97	2.47	3.342 (4)	149
C26—H26A···O3^i^	0.97	2.47	3.373 (3)	155

Symmetry codes:  (i) *x*−1, *y*, *z*.
